# The impact of relative dose intensity on pathological complete response in neoadjuvant chemotherapy of muscle-invasive urothelial cancer: a multicenter retrospective study

**DOI:** 10.1093/oncolo/oyag256

**Published:** 2026-07-02

**Authors:** Giuseppe Neola, Fabiano Flauto, Carmine Caso, Giuseppina Montuori, Marco Maruzzo, Eleonora Lai, Giuseppe Luigi Banna, Michele Maffezzoli, Mimma Rizzo, Francesco Massari, Veronica Mollica, Sarah Scagliarini, Vincenza Conteduca, Patrizia Giannatempo, Alessandro Rametta, Brigida Anna Maiorano, Gaetano Facchini, Edoardo Lenci, Rosa Tambaro, Francesco Grillone, Davide Bosso, Felice Crocetto, Ciro Imbimbo, Alberto Servetto, Roberto Bianco, Luigi Formisano

**Affiliations:** Department of Clinical Medicine and Surgery, Federico II University, Via Sergio Pansini, 5, Naples, 80131, Italy; Portsmouth Hospitals University NHS Trust, Portsmouth, PO63LY, UK; Department of Clinical Medicine and Surgery, Federico II University, Via Sergio Pansini, 5, Naples, 80131, Italy; Department of Clinical Medicine and Surgery, Federico II University, Via Sergio Pansini, 5, Naples, 80131, Italy; Department of Clinical Medicine and Surgery, Federico II University, Via Sergio Pansini, 5, Naples, 80131, Italy; Oncology Unit 3, Istituto Oncologico Veneto - IOV IRCCS, Padova, 35128, Italy; Oncology Unit 3, Istituto Oncologico Veneto - IOV IRCCS, Padova, 35128, Italy; Portsmouth Hospitals University NHS Trust, Portsmouth, PO63LY, UK; Faculty of Science & Health, School of Pharmacy & Biomedical Sciences, University of Portsmouth, Portsmouth, PO63LY, UK; Department of Medicine and Surgery, University Hospital of Parma—Medical Oncology Unit, University of Parma, Parma, 43126, Italy; Medical Oncology Unit, Azienda Ospedaliera Universitaria Consorziale Policlinico di Bari, Bari, 70124, Italy; Medical Oncology, IRCCS Azienda Ospedaliero-Universitaria di Bologna, Bologna, 40138, Italy; Medical Oncology, IRCCS Azienda Ospedaliero-Universitaria di Bologna, Bologna, 40138, Italy; UOC di Oncologia, Azienda Ospedaliera di Rilievo Nazionale Cardarelli di Napoli, Naples, 80131, Italy; Unit of Medical Oncology and Biomolecular Therapy, Department of Medical and Surgical Sciences, University of Foggia, Policlinico Riuniti, 71122, Italy; Genitourinary Medical Oncology—Fondazione IRCCS Istituto Nazionale dei Tumori di Milano, Milan, 80131, Italy; Genitourinary Medical Oncology—Fondazione IRCCS Istituto Nazionale dei Tumori di Milano, Milan, 80131, Italy; Department of Medical Oncology, IRCCS San Raffaele Hospital, Milan, 20132, Italy; Oncology Unit, S. Maria Delle Grazie Hospital, Pozzuoli, Naples, 80078, Italy; Medical Oncology Unit, Azienda Ospedaliera Ospedali Riuniti Marche Nord, Pesaro, 61121, Italy; Uro-Gynecological Oncology, Istituto Nazionale Tumori—IRCCS—Fondazione G. Pascale, Naples, 80131, Italy; SOC Oncologia, PO Pugliese-Ciaccio, Azienda Ospedaliera Universitaria Renato Dulbecco, Catanzaro, 88100, Italy; Medical Oncology Unit, Ospedale del Mare, Naples, 80147, Italy; Department of Neurosciences, Reproductive Sciences and Odontostomatology, University of Naples Federico II, Naples, 80131, Italy; Department of Neurosciences, Reproductive Sciences and Odontostomatology, University of Naples Federico II, Naples, 80131, Italy; Department of Clinical Medicine and Surgery, Federico II University, Via Sergio Pansini, 5, Naples, 80131, Italy; Department of Clinical Medicine and Surgery, Federico II University, Via Sergio Pansini, 5, Naples, 80131, Italy; Department of Clinical Medicine and Surgery, Federico II University, Via Sergio Pansini, 5, Naples, 80131, Italy

**Keywords:** urothelial cancer, neoadjuvant, relative dose intensity, pathological complete response, bladder cancer

## Abstract

**Background:**

Cisplatin-based neoadjuvant chemotherapy (NAC) improves survival in muscle-invasive urothelial cancer (MIUC), yet pathological complete response (pCR) is achieved in a minority of patients. Relative dose intensity (RDI) is a potentially modifiable determinant of chemotherapy efficacy, but its impact during NAC in MIUC remains unexplored.

**Methods:**

This multicenter retrospective study included patients with MIUC treated with cisplatin–gemcitabine NAC followed by radical cystectomy. Relative dose intensity was calculated from original administration records and categorized as ≥85% vs <85%. The primary endpoint was pCR (ypT0N0M0). Secondary endpoints included overall survival (OS) and event-free survival (EFS).

**Results:**

A total of 330 patients were included; 66% maintained RDI ≥ 85%. Overall pCR rate was 25.2%. Patients with RDI ≥ 85% achieved higher pCR rates compared with those with RDI < 85% (29.7% vs 16.2%; *P* = .008). Relative dose intensity  ≥ 85% remained independently associated with pCR in multivariable analysis (odds ratio = 2.10; *P* = .032). Preserved RDI was also associated with improved EFS (hazard ratio [HR] = 0.65; *P* = .039) and OS (HR = 0.53; *P* = .026) at univariate analysis. Achievement of pCR was strongly associated with superior EFS (HR = 0.23; *P* < .001) and OS (HR = 0.35; *P* = .009).

**Conclusion:**

Preservation of cisplatin RDI ≥ 85% during NAC is independently correlated with higher pCR rates and associated to a better survival trend in MIUC. Optimization of chemotherapy delivery represents a clinically actionable strategy to enhance neoadjuvant efficacy.

Implications for Practice Among patients with muscle-invasive urothelial cancer receiving neoadjuvant cisplatin–gemcitabine chemotherapy, preservation of cisplatin relative dose intensity (RDI) ≥85% was associated with higher pathological complete response rates and numerically improved survival outcomes. These findings suggest that chemotherapy delivery should be carefully monitored and optimized whenever clinically feasible. Although causality cannot be established because of the retrospective design, RDI may represent a practical treatment-delivery metric that could help identify patients at risk of suboptimal outcomes and inform future prospective studies.

## Introduction

Muscle-invasive urothelial cancer (MIUC) is an aggressive and biologically heterogeneous malignancy characterized by a substantial risk of early systemic dissemination. Cisplatin-based (CB) neoadjuvant chemotherapy (NAC), offered for 3-4 cycles, followed by radical cystectomy, represents the established standard of care and offers a significant overall survival (OS) improvement compared with upfront surgery.^1–4^ Despite this proven benefit, the therapeutic performance of NAC remains suboptimal. To date, pathological complete response (pCR) is the only validated surrogate of long-term survival and an indicator of chemosensitivity; however, only 25%-30% of patients achieve this endpoint with standard chemotherapy.^2^^,^^3^ The recent phase III EV-303 and NIAGARA trials further strengthened the prognostic value of pCR, demonstrating that patients who achieve a pCR have significantly improved event-free survival (EFS) and OS compared with those with residual disease at cystectomy.^4^^,^^5^

Relative dose intensity (RDI) has emerged as a potential clinically meaningful metric. Relative dose intensity reflects the ratio between the delivered chemotherapy dose intensity and the planned dose intensity over the intended time frame. Across several malignancies, including triple-negative breast cancer, gastroesophageal cancers, lung cancer, and pancreatic cancer, maintaining an RDI value ≥85% has been consistently associated with higher rates of pCR and improved patient outcomes.^6–11^ Conversely, reductions in RDI have been associated with lower treatment efficacy and inferior oncologic outcomes in several malignancies.^12^ The relevance of maintaining optimal dose delivery is particularly evident in curative-intent and perioperative settings, where chemotherapy must be administered at adequate intensity to promote meaningful tumor regression and avoid disease relapse.

Despite extensive evidence supporting the role of RDI in other solid tumors, to our knowledge its impact in NAC of MIUC has never been investigated.^13^ Therefore, whether maintaining higher RDI during NAC is associated with higher pCR rates and improved survival outcomes remains an unmet need in the current literature. Given that RDI is directly influenced by clinical decision-making, toxicity management, and supportive care strategies, elucidating its role may provide clinicians with critical insight into how dose reductions or treatment delays during NAC may potentially compromise oncologic outcomes in MIUC.

In light of these considerations, the present study aimed to evaluate the association between RDI of cisplatin plus gemcitabine (CG) NAC and survival outcomes in MIUC, and to investigate whether reduced RDI is associated with worse survival outcomes.

## Methods

### Trial oversight

This retrospective, multicenter cohort study was conducted across academic urological oncology units in Italy. The study protocol, including all analytical procedures and subsequent amendments, received central approval from the local ethical committee. The study complied with the Declaration of Helsinki, Good Clinical Practice principles, and General Data Protection Regulation regulations. Clinical datasets were anonymized before transfer to the coordinating center.

Informed consent for retrospective data use was obtained where required by local legislation, while a consent exemption was granted at centers where permitted.

### Study design

The investigation was a multicenter, retrospective, observational study. After data collection, each patient’s RDI value was calculated from original chemotherapy administration records according to the formula described in section RDI definition.

Based on the adopted definitions in the oncologic literature, a cisplatin RDI threshold of 85% was prespecified at the time of study design to define RDI cutoff. Accordingly, patients were assigned to two prespecified cohorts based on cisplatin RDI values: arm A (RDI ≥ 85%) and arm B (RDI < 85%). No randomization or protocol-mandated intervention was performed.

To detect an absolute difference of 10% in pCR rates between the study groups, in line with clinically meaningful differences observed in contemporary chemotherapy-based neoadjuvant trials such as NIAGARA,^5^ assuming a two-sided α of 0.05 and 80% statistical power, a total sample size of approximately 300 patients was estimated to be required.

Clinical and pathological outcomes were then evaluated and compared between the two RDI-defined groups in accordance with the statistical analysis plan.

### Inclusion and exclusion criteria

The study included consecutive patients treated between 2015 and 2025 who received NAC with CG followed by radical cystectomy.

Eligible patients were adults (≥18 years) with histologically confirmed MIUC and a clinical stage of T2-T4a, N0-N+, M0, according to the eighth edition of the AJCC Cancer Staging Manual.^14^ All patients were required to be eligible for CG chemotherapy, with a creatinine clearance ≥ 60 mL/min per 1.73 m^2^, and to be medically fit to undergo radical cystectomy. Eligibility was restricted to patients who, upon completion of NAC, proceeded to radical cystectomy as definitive local treatment. Patients managed with alternative local strategies after NAC, including radiotherapy or transurethral resection of the bladder tumor, were excluded from the study. Non-urothelial histology was allowed only if accounted for less than 50% of the histological specimen obtained from radical cystectomy.

Other exclusion criteria included metastatic disease at baseline, prior systemic therapy for urothelial carcinoma, uncontrolled comorbidities precluding cisplatin administration, concurrent active malignancies requiring systemic treatment, pregnancy, and incomplete documentation of chemotherapy dosing or scheduling parameters. Only patients who completed at least three cycles of CG of neoadjuvant treatment as per standard of care were included, to ensure accurate estimation of dose intensity and to avoid distortion related to incomplete treatment or shortened treatment intervals.

Demographic, clinical, radiologic, and pathological data were prospectively recorded in a harmonized electronic case report form. Collected variables included age, sex, body mass index, Charlson Comorbidity Index (CCI), smoking status, presence of hydronephrosis or nephrostomy, baseline clinical staging, histologic subtype, renal function, interval between completion of NAC and surgery, and complete NAC delivery parameters, including planned and administered doses, treatment dates, intervals, and delays. Operative details and postoperative pathological findings were retrieved from electronic medical records.

**Figure 1.**
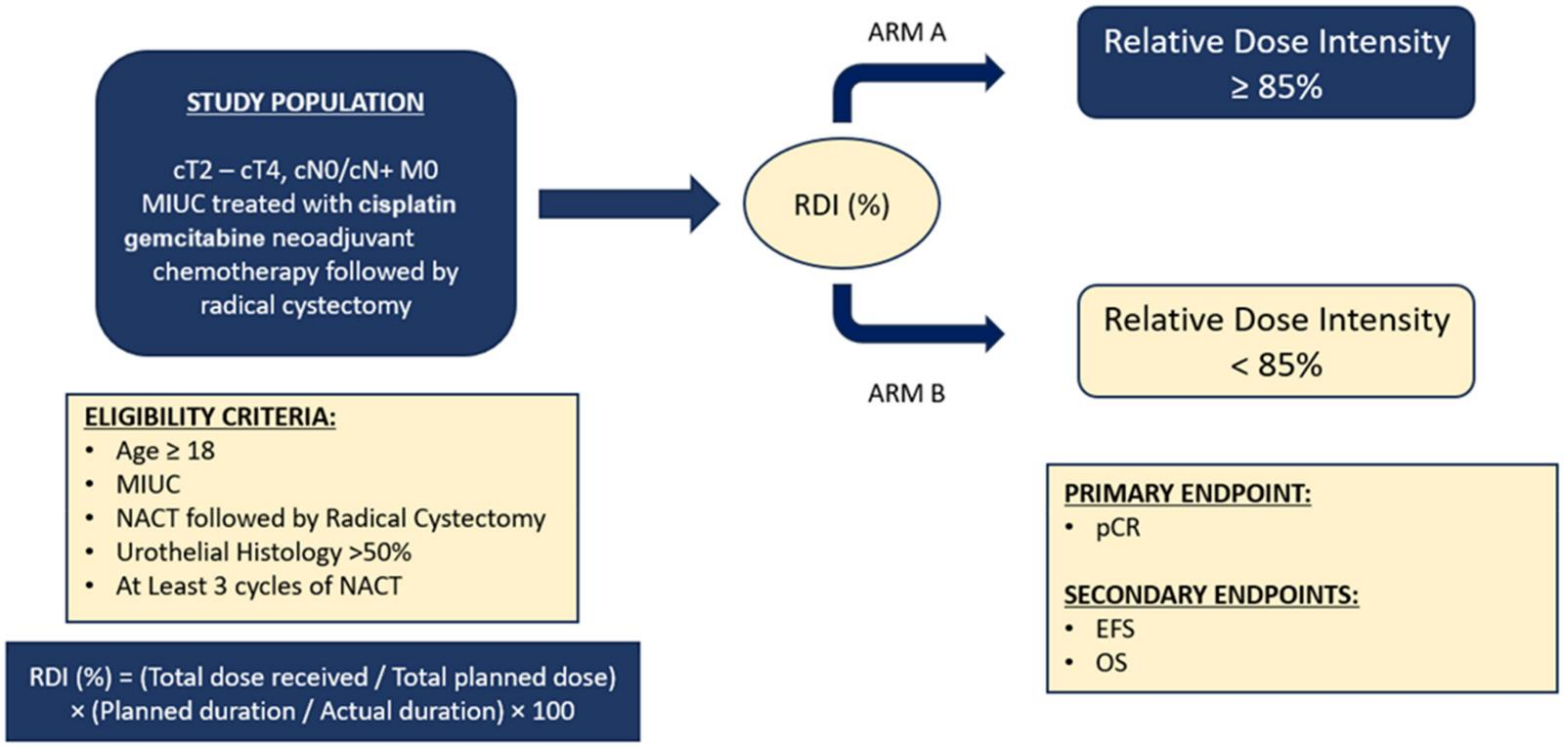
Study design.

A schematic overview of the study design is provided in Figure 1.

### RDI definition

Relative dose intensity was defined as the amount of drug administered (mg/m^2^ of Body Surface Area) per unit of time (days) relative to the planned dose intensity. The planned intensity corresponded to cisplatin 70 mg/m^2^ administered every 21 days. Relative dose intensity was thus calculated using the following formula:

RDI (%) = (Total dose received/Total planned dose) × (Planned duration/Actual duration) × 100.

The total dose reflected the cumulative cisplatin dose received (mg) regardless of the time intervals, while planned and actual durations represented the ideal and real-time intervals between the first and last chemotherapy administration, respectively. Relative dose intensity was analyzed as a categorical variable using a prespecified threshold of ≥85%.^15–17^

### Endpoint assessment

The primary endpoint was pCR rate, defined as the proportion of patients with a final pathological stage of ypT0N0M0 based on cystectomy specimens obtained after NAC completion. Pathological response was assessed by local pathology review in accordance with the AJCC/TNM eighth edition staging system.^14^ Patients with residual carcinoma in situ (ypTis) were not considered to have achieved pCR.

Secondary endpoints included OS and EFS. Overall survival was defined as the time interval in months from NAC initiation to death from any cause regardless of whether the patient had received subsequent anticancer therapy. Event-free survival was defined as the time interval in months from NAC initiation to progressive disease, the first recurrence of disease after radical cystectomy or death from any cause. A recurrence of disease includes local pelvic recurrence of urothelial carcinoma (UC), urinary tract recurrence of UC, or distant metastasis of UC. Radiologic tumor assessments were completed in each participating center referring to the Response Evaluation Criteria in Solid Tumors, version 1.1. Disease recurrence and survival status were ascertained from electronic medical records and national health registries.

### Statistical analysis

Statistical analyses were conducted according to a prespecified plan with the objective of evaluating the association between RDI and pathological response and survival outcomes. Baseline demographic, clinical, and pathological characteristics were summarized using conventional descriptive methods.

Subsequently, univariate binomial logistic regression was performed to quantify the strength of the association and estimate odds ratios, which constituted the primary analytic approach for this endpoint. To evaluate the consistency of the RDI effect, an exploratory multivariable model was constructed including clinically relevant covariates such as clinical T and N stage, smoking status, histology, Eastern Cooperative Oncology Group (ECOG) performance status, comorbidity burden, and the interval between completion of chemotherapy and surgery. For the multivariable logistic regression model evaluating pCR, covariates were selected a priori based on clinical relevance and established prognostic value. The model included RDI group, clinical T stage, clinical N stage, ECOG performance status, CCI, smoking status, histology, and time from NAC completion to radical cystectomy. Multicollinearity was evaluated using variance inflation factors, with values <5 considered indicative of no relevant multicollinearity. Model discrimination was assessed using the area under the receiver operating characteristic curve derived from predicted probabilities. Calibration was assessed using the Hosmer–Lemeshow goodness-of-fit test, with *P* > .05 interpreted as no evidence of poor fit.

Survival outcomes were analyzed using time-to-event methods. The OS and EFS were estimated using the Kaplan–Meier method, and differences between RDI groups were evaluated with the log-rank test. Univariate Cox proportional hazards models were fitted to estimate hazard ratios and corresponding confidence intervals. Multivariable Cox proportional hazards models were then constructed for both OS and EFS to adjust for clinically relevant covariates selected a priori based on clinical relevance and established prognostic value. The models included RDI group, clinical T stage, clinical N stage, ECOG performance status, pCR, histology, CCI, smoking status, and time from completion of NAC to radical cystectomy, dichotomized as >8 weeks vs ≤8 weeks. To minimize confounding from post-neoadjuvant treatments, patients who received adjuvant systemic therapy were excluded from the EFS analysis, in accordance with the statistical analysis plan.

Exploratory analyses assessed the prognostic implications of pCR. The OS and EFS were estimated using the Kaplan–Meier curves stratified by pCR status, and between-group differences were evaluated using the log-rank test. The median follow-up time was calculated using the reverse Kaplan–Meier method to account for censored data.

As an exploratory sensitivity analysis, RDI was additionally analyzed as a continuous variable to evaluate potential dose–response relationships between chemotherapy exposure and pathological response. Logistic regression models were fitted using RDI expressed per 10% increase.

Treatment-related adverse events were summarized according to the RDI group and compared using Fisher’s exact test, as appropriate for sparse categorical data. Toxicities were reported according to CTCAE v5.0 and grouped as grade 1-2 and grade 3-4 events.

Sensitivity analyses stratified by ECOG performance status and CCI were performed to evaluate the consistency of the association between RDI and pCR across clinically relevant subgroups.

All statistical analyses were conducted using Jamovi (version 2.3.28.0),^18^ and all graphical outputs, including swimmer plots and survival curves, were generated using R (version 4.4.2).^19–21^

## Results

### Patient baselines characteristics

Between September 2015 and September 2025, 330 patients met the eligibility criteria and were included in the final analysis. The achieved sample size met the prespecified requirement to ensure adequate statistical power. Baseline demographic, clinical, and pathological characteristics are summarized in Table 1.

**Table 1:** Baseline demographic, clinical, and pathological characteristics of the overall population stratified according to RDI group. Percentages are calculated within each group.

Characteristics	Population (*N* = 330)	RDI high (*N* = 219)	RDI low (*N* = 111)
**Sex**	Male: 248 (75%)	Male: 162 (74%)	Male: 86 (77%)
	Female: 82 (25%)	Female: 57 (26%)	Female: 25 (23%)
**ECOG performance status**	0: 275 (83%)	0: 190 (87%)	0: 85 (77%)
	1: 45 (14%)	1: 23 (10%)	1: 22 (20%)
	2: 10 (3%)	2: 6 (3%)	2: 4 (3%)
**Smoker status**	Nonsmoker: 71 (21%)	Nonsmoker: 42 (19%)	Nonsmoker: 29 (26%)
	Former: 136 (41%)	Former: 89 (41%)	Former: 47 (42%)
	Current: 123 (38%)	Current: 88 (40%)	Current: 35 (32%)
**Charlson Comorbidity index**	CCI 0-2: 85 (25%)	CCI 0-2: 53 (24%)	CCI 0-2: 32 (29%)
	CCI 3-4: 124 (38%)	CCI 3-4: 83 (38%)	CCI 3-4: 41 (37%)
	CCI >5: 121 (37%)	CCI >5: 83 (38%)	CCI >5: 38 (34%)
**Clinical T stage**	cT1 diverticulum: 4 (1%)	cT1 diverticulum: 2 (1%)	cT1 diverticulum: 2 (2%)
	cT2: 256 (78%)	cT2: 167 (76%)	cT2: 89 (80%)
	cT3: 61 (18%)	cT3: 43 (20%)	cT3: 18 (16%)
	cT4: 9 (3%)	cT4: 7 (3%)	cT4: 2 (2%)
**Clinical N stage**	cN0: 279 (84%)	cN0: 187 (85%)	cN0: 92 (83%)
	cN+: 51 (16%)	cN+: 32 (14%)	cN+: 19 (17%)
**Tumor histology**	Urothelial: 275 (83%)	Urothelial: 180 (82%)	Urothelial: 95 (85%)
	Non-urothelial differentiation: 55 (17%)	Non-urothelial differentiation: 39 (18%)	Non-urothelial differentiation: 16 (15%)
**Pretreatment nephrostomy**	No: 291 (89%)	No: 198 (91%)	No: 93 (84%)
	Unilateral: 15 (4%)	Unilateral: 9 (4%)	Unilateral: 6 (5%)
	Bilateral: 7 (2%)	Bilateral: 5 (2%)	Bilateral: 2 (2%)
	Missing: 17 (5%)	Missing: 7 (3%)	Missing: 10 (9%)
**Pretreatment hydronephrosis**	None: 276 (85%)	None: 187 (85%)	None: 89 (80%)
	Unilateral: 44 (12%)	Unilateral: 26 (12%)	Unilateral: 18 (16%)
	Bilateral: 10 (3%)	Bilateral: 6 (3%)	Bilateral: 4 (4%)
**Platinum cycles number**	3 cycles: 153 (46%)	3 cycles: 101 (46%)	3 cycles: 53 (48%)
	4 cycles: 177 (54%)	4 cycles: 118 (54%)	4 cycles: 58 (52%)
**Pathological tumor stage (ypT)**	ypT0: 83 (25%)	ypT0: 65 (30%)	ypT0: 18 (16%)
	ypTis: 52 (16%)	ypTis: 43 (20%)	ypTis: 9 (8%)
	ypT1: 39 (11%)	ypT1: 27 (12%)	ypT1: 12 (11%)
	ypT2: 59 (18%)	ypT2: 22 (10%)	ypT2: 37 (33%)
	ypT3: 68 (21%)	ypT3: 40 (18%)	ypT3: 28 (25%)
	ypT4: 29 (9%)	ypT4: 22 (10%)	ypT4: 7 (6%)
**Grading**	G1: 21 (6%)	G1: 13 (6%)	G1: 8 (7%)
	G2: 23 (7%)	G2: 12 (5%)	G2: 11 (10%)
	G3: 286 (87%)	G3: 194 (89%)	G3: 92 (83%)
**Surgical residual margin**	R0: 311 (94%)	R0: 207 (94.5%)	R0: 103 (93%)
	R1: 9 (2.7%)	R1: 5 (2.3%)	R1: 4 (3.6%)
	R2: 2 (0.6%)	R2: 0	R2: 2 (1.7%)
	Missing: 9 (2.7%)	Missing: 7 (3.2%)	Missing: 2 (1.7%)
**Time to surgery**	<6 weeks: 98 (30%)	<6 weeks: 55 (25%)	<6 weeks: 43 (39%)
	6-8 weeks: 73 (22%)	6-8 weeks: 45 (21%)	6-8 weeks: 28 (25%)
	>8 weeks: 159 (48%)	>8 weeks: 119 (54%)	>8 weeks: 40 (36%)
**Adjuvant anticancer treatment**	No: 320 (97%)	No: 211 (96%)	No: 109 (98%)
	Yes: 10 (3%)	Yes: 8 (4%)	Yes: 2 (2%)

Abbreviation: RDI, relative dose intensity.

The cohort was predominantly male (248 patients, 75%). Most patients had good performance status, with 275 (83%) presenting an ECOG performance status of 0 and 55 (17%) having an ECOG performance status of 1-2. Tobacco exposure was common, with 123 patients (38%) being current smokers and 136 (41%) former smokers. A moderate-to-high comorbidity burden was observed in most patients, with 245 individuals (74%) presenting a CCI ≥ 3.

At clinical staging, 256 patients (78%) had cT2 tumors, whereas 70 patients (21%) presented cT3-T4a disease. Clinical nodal involvement was observed in 51 patients (16%). Pure urothelial carcinoma accounted for most cases (275 patients, 83%), while variant differentiation was identified in 55 patients (17%). Pretreatment hydronephrosis was present in 54 patients (15%), and a nephrostomy was required in 22 patients (7%).

Overall, 219 patients (66.4%) maintained a RDI ≥85%, whereas 111 patients (33.6%) received an RDI < 85%. Three cycles of NAC were administered in 153 patients (46%), while 177 patients (54%) completed four cycles.

**Figure 2.**
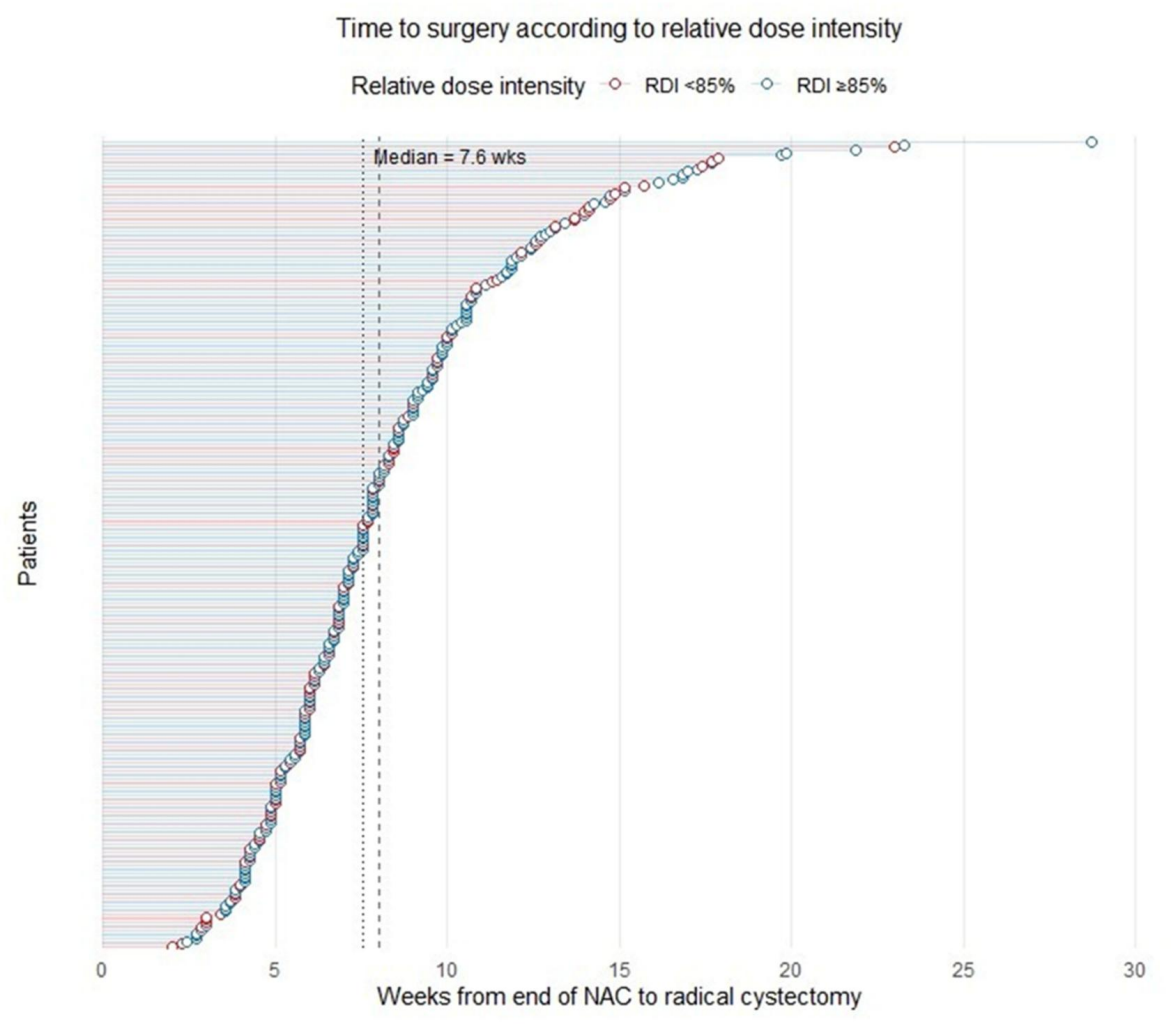
Swimmer plot illustrating the interval between completion of NAC and radical cystectomy for individual patients. Each horizontal line represents a single patient, ordered by increasing time to surgery, with the marker indicating the timing of cystectomy. Vertical dashed lines indicate prespecified time thresholds, and the dotted line denotes the median time to surgery (7.6 weeks) for the overall cohort. NAC, neoadjuvant chemotherapy.

The interval between completion of NAC and radical cystectomy was <6 weeks in 98 patients (30%), 6-8 weeks in 73 patients (22%), and >8 weeks in 159 patients (48%). The median interval between the last NAC administration and surgery was 7.6 weeks. The distribution of time to surgery largely overlapped between patients with RDI ≥ 85% and those with RDI < 85% (Figure 2).

At cystectomy, negative surgical margins were achieved in 311 patients (94%). Adjuvant systemic therapy was administered in 10 patients (3%), including 8 patients in the RDI-high group and 2 in the RDI-low group.

Treatment-related adverse events are summarized in Table S1. Overall, toxicity profiles were broadly comparable between RDI groups. Grade 1-2 neutropenia and nausea were more frequently observed among patients maintaining RDI ≥85% compared with those receiving RDI <85% (32.4% vs 19.8%, *P* = .020; and 29.2% vs 14.4%, *P* = .003, respectively). Similarly, grade 3-4 neutropenia was more frequent in the RDI-high group (18.7% vs 9.0%, *P* = .028). No statistically significant differences were observed for the remaining grade 3-4 toxicities.

### pCR rate

**Figure 3.**
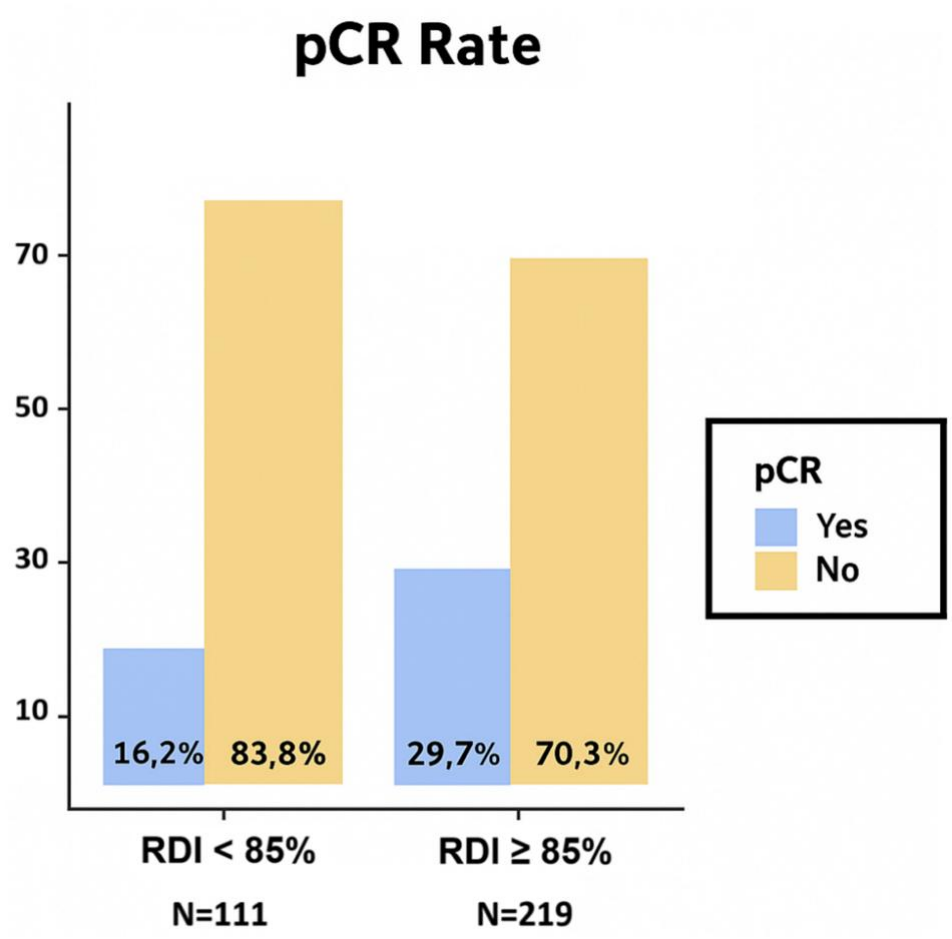
pCR rate according to relative dose intensity group. pCR, pathological complete response.

The overall pCR rate in the study cohort was 25.2% (83/330). Patients who maintained an RDI ≥85% achieved pCR in 29.7% of cases (65/219), compared with 16.2% (18/111) among those with an RDI <85% (χ^2^ test *P* = .008) (Figure 3). In univariate logistic regression analysis, an RDI ≥85% was significantly associated with higher odds of achieving pCR (odds ratio [OR] 2.20, 95% CI, 1.22-3.90; *P* = .009).

In an exploratory multivariable logistic regression model adjusting for clinically relevant baseline covariates, RDI ≥ 85% remained associated with higher odds of achieving pCR (adjusted OR 2.1, 95% CI, 1.07-4.08; *P* = .032). Clinical nodal involvement was also associated with pCR, with cN+ disease showing lower odds of pCR compared with cN0 status (adjusted OR 0.23, 95% CI 0.06-0.80; *P* = 0.021). In addition, nonsmokers had higher odds of pCR compared with current smokers (adjusted OR 2.75, 95% CI 1.31-5.78; *P* = .008). No significant associations were observed for the remaining covariates (Table S2).

No missing data were present in the covariates included in the analysis. No relevant multicollinearity was detected among the covariates included in the pCR multivariable logistic regression model, with VIF values ranging from 1.02 to 1.05. The model showed acceptable discriminatory ability for pCR prediction, with an area under curve (AUC) of 0.733. Calibration was assessed using the Hosmer–Lemeshow goodness-of-fit test, which showed no evidence of poor fit (χ^2^=13.92, df = 8, *P* = .084).

In the exploratory sensitivity analysis treating RDI as a continuous variable, each 10% increase in RDI was associated with numerically higher odds of achieving pCR, although this association did not reach statistical significance (OR 1.10, 95% CI 0.96-1.26; *P* = .175) (Table S3).

Sensitivity analyses stratified by ECOG performance status and CCI demonstrated a consistent direction of association between RDI ≥85% and higher pCR rates across clinically relevant subgroups, although statistical precision was reduced in smaller strata (Table S4).

### Survival outcomes

Event-free survival analysis included 320 patients after excluding individuals who received adjuvant systemic therapy to avoid confounding from post-surgical treatments. During follow-up, 59 events (27.0%) occurred in arm A and 36 events (32.4%) in arm B, out of 211 and 109 patients, respectively. The median duration of follow-up for EFS was 26.6 months (range 3.1-124.4) (Figure S1). Median EFS was not reached in either group.

**Figure 4.**
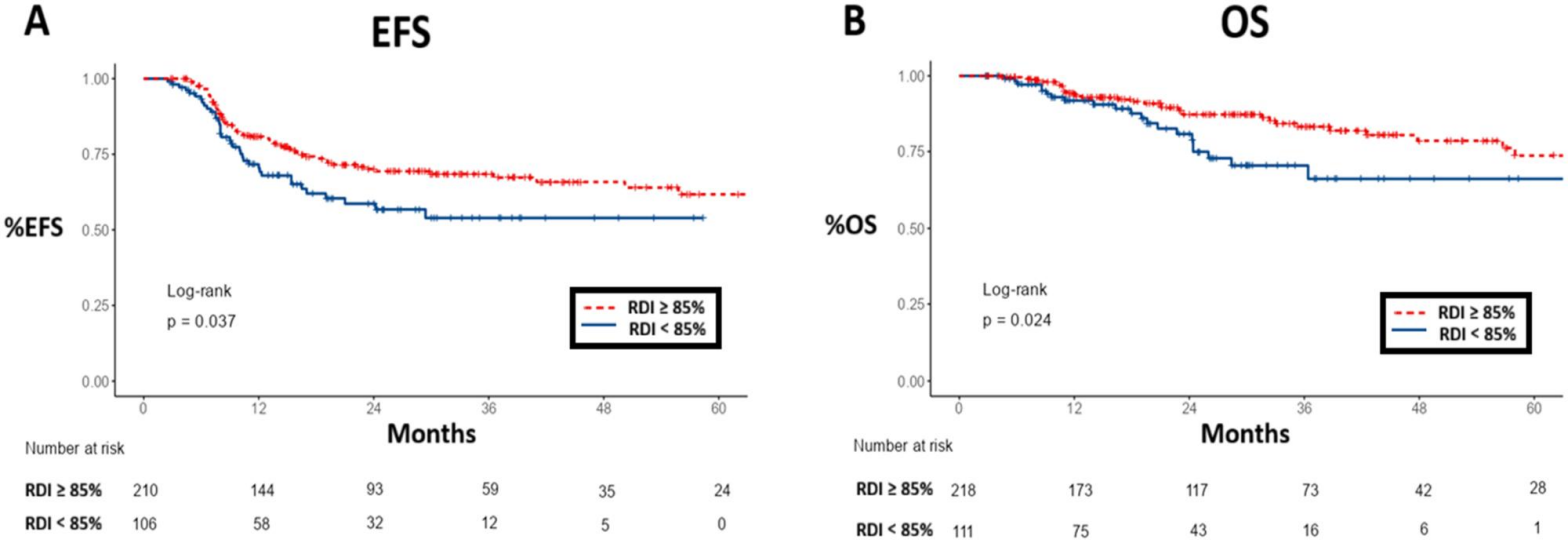
(A) EFS and (B) OS stratified by RDI levels. Tick marks indicate censored observations, and numbers at risk are shown below the plot. EFS, event-free survival; OS, overall survival; RDI, relative dose intensity.

The log-rank test confirmed a significant difference in EFS comparisons among arms A and B (*P* = .037). In univariate Cox regression, maintaining RDI ≥ 85% was associated with a significantly lower risk of recurrence, progression, or death compared with RDI < 85% (hazard ratio [HR] = 0.65; 95% CI, 0.43-0.98; *P* = .039) (Figure 4). After adjustment for clinically relevant covariates, including clinical T and N stage, ECOG performance status, pathological complete response, histology, CCI, time to surgery > 8 weeks, and smoking status, RDI ≥85% was associated with a numerically lower risk of EFS events, although this association did not reach the statistical significance (adjusted HR = 0.76; 95% CI, 0.48-1.22; *P* = .254).

Patients in the RDI-high cohort demonstrated estimated EFS probabilities of 80.8% (95% CI, 75.47%-86.5%) at 12 months, 68.4% (95% CI, 61.82%-75.8%) at 36 months, and 61.7% (95% CI, 53.37%-71.4%) at 60 months, whereas the RDI-low group exhibited EFS rates of 70.4% (95% CI, 61.72%-80.3%), 53.9% (95% CI, 43.23%-67.1%), and 53.9% (95% CI, 43.23%-67.1%), respectively. The identical 36 and 60 month estimates in the RDI-low group reflect the absence of additional events beyond month 36, resulting in a survival plateau.

Death occurred in 32/219 patients (14.6%) in the RDI-high group and in 21/111 patients (18.9%) in the RDI-low group. The median duration of follow-up for OS was 26.6 months (range 3.1-124.4) (Figure S2). Median OS was not reached in either cohort during the observation period.

The Kaplan–Meier analysis demonstrated a consistent and progressive separation of survival curves in favor of patients who maintained RDI ≥85%. The difference between groups was statistically significant (log-rank *P* = .024). In univariate Cox regression, maintaining RDI ≥85% was associated with a significantly lower risk of death compared with RDI <85% (HR = 0.53; 95% CI, 0.30-0.93; *P* = .026) (Figure 4). After adjustment for clinically relevant covariates, including clinical T and N stage, ECOG performance status, pathological complete response, histology, CCI, time to surgery > 8 weeks and smoking status, RDI ≥85% showed a trend toward improved OS, although this association narrowly missed statistical significance (adjusted HR = 0.57; 95% CI, 0.30-1.08; *P* = .085).

Estimated OS at 12, 36, and 60 months for the RDI-high group was 93.9% (95% CI, 90.6%-97.3%), 83.2% (95% CI, 77.2%-89.6%), and 73.6% (95% CI, 64.4%-84.2%), respectively. The corresponding survival probabilities in the RDI-low cohort were 91.8% (95% CI, 86.5%-97.4%), 70.4% (95% CI, 59.6%-83.1%), and 66.0% (95% CI, 53.5%-81.3%).

### pCR and survival

Exploratory survival analyses stratified by pCR demonstrated marked differences in outcomes between patients achieving pCR and those with residual disease.

In univariable Cox regression analysis, pCR status after NAC emerged as a strong prognostic factor for EFS. Patients achieving pCR had a 77% relative reduction in the risk of EFS events compared with non-pCR patients (HR = 0.23, 95% CI, 0.12-0.45; *P* < 0.001).

Median EFS was not reached among patients with pCR compared with 14.4 months in those without pCR. The Kaplan–Meier curves revealed substantial divergence between the two groups, with significantly improved EFS for patients achieving pCR (log-rank *P* < 0.0001) (Figure 4).

Accordingly, univariable Cox regression analysis showed that patients achieving pCR after NAC experienced a 65% relative reduction in the risk of death compared with non-pCR patients (HR = 0.35, 95% CI, 0.16-0.77; *P* = .009).

**Figure 5.**
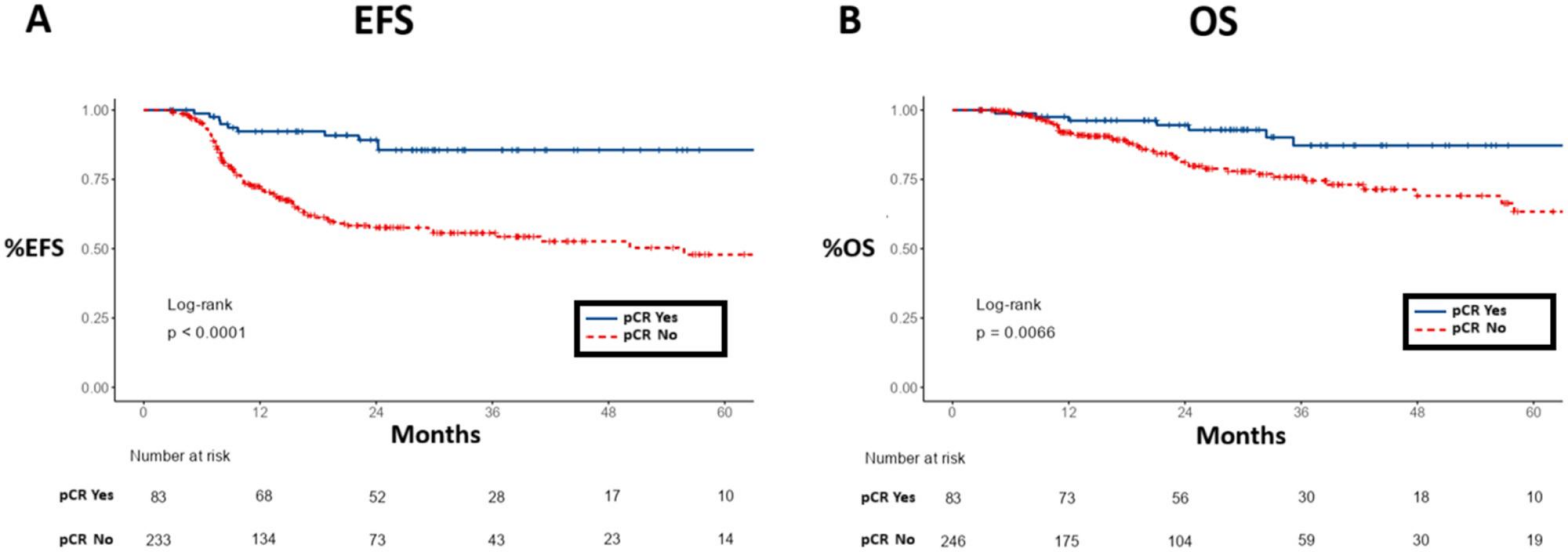
(A) EFS and (B) OS stratified by pCR. Tick marks indicate censored observations, and numbers at risk are shown below the plot. EFS, event-free survival; OS, overall survival; pCR, pathological complete response; RDI, relative dose intensity.

Median OS was not reached in either group, yet the Kaplan–Meier curves showed clear and progressive separation, with patients achieving pCR experiencing significantly longer OS (log-rank *P* = .0066) (Figure 5).

## Discussion

In this multicenter retrospective analysis, preservation of cisplatin RDI ≥85% during NAC was independently associated with a higher probability of achieving pCR and showed a numerical association with improved survival outcomes in patients with MIUC. Beyond confirming the established prognostic relevance of pCR, these findings suggest that treatment delivery may represent a clinically relevant and potentially modifiable factor associated with neoadjuvant efficacy in contemporary practice.^22^^,^^23^

The association between RDI and pathological response represents the central observation of this study. Patients who maintained at least 85% of the planned cisplatin dose intensity achieved nearly double the pCR rate compared with those treated at lower intensity. This effect size is clinically relevant and biologically plausible, given the well-established dose–response relationship of platinum compounds and the dependence of cytotoxic efficacy on cumulative exposure and schedule adherence.^24^ Comparable associations between reduced dose intensity and impaired pathological response have been consistently reported across multiple solid tumors, including triple-negative breast cancer and upper gastrointestinal malignancies, where preservation of planned chemotherapy intensity has translated into higher rates of tumor eradication.^25^ Conversely, evidence in NAC in urothelial carcinoma has historically been limited to cumulative dose analyses in the MVAC era, without a formal evaluation of RDI using contemporary cisplatin–gemcitabine regimens.^26–28^ Our findings, therefore, fill a critical gap by demonstrating that even within standard-of-care NAC, deviations from planned dose intensity may meaningfully compromise pathological response.

Importantly, the association between higher RDI and increased likelihood of pCR remained robust after adjustment for baseline clinical and pathological covariates in multivariable logistic regression. The persistence of statistical significance in the adjusted model supports the robustness of the association between higher RDI and pCR, although residual confounding inherent to the retrospective design cannot be fully excluded. Among the covariates included in the model, clinical nodal involvement emerged as an independent negative predictor of pCR, a finding that is biologically expected given the greater tumor burden and higher likelihood of micro metastatic dissemination.^29^^,^^30^ Smoking status also retained independent prognostic significance, consistent with prior evidence linking active tobacco exposure to impaired chemotherapy response.^31^ The absence of significant associations for other clinical variables further supports the robustness of the observed association between dose intensity and pathological response.

Survival analyses reinforce the clinical relevance of these findings. Patients maintaining RDI ≥85% exhibited higher OS and EFS probabilities throughout follow-up at univariate analysis, with differences emerging early and persisting over time. Although adjusted survival analyses did not retain statistical significance after multivariable correction, the observed association between preserved dose intensity, higher pCR rates, and improved survival outcomes suggests a biologically plausible relationship between chemotherapy exposure and disease control.^32^ This interpretation is further supported by exploratory analyses stratified by pCR, which demonstrated markedly superior OS and EFS among patients achieving pCR compared with those with residual disease.

The prognostic patterns observed in our cohort closely mirror those reported in contemporary phase III perioperative trials, most notably NIAGARA^5^ and EV-303.^4^ In NIAGARA, the addition of durvalumab to CB perioperative chemotherapy increased pCR rates from approximately 27%-28% with chemotherapy alone to nearly 37%-38% in the experimental arm, with corresponding improvements in EFS and OS.^5^ Notably, patients achieving pCR in NIAGARA experienced early and sustained separation of survival curves, validating the central role of pCR as a surrogate of long-term outcomes. A highly consistent prognostic profile has been reported in EV-303,^4^ where perioperative strategies incorporating enfortumab vedotin and pembrolizumab similarly demonstrated that achievement of pCR was associated with substantially improved EFS and OS. Together, these trials firmly establish pCR as a reliable surrogate endpoint in muscle-invasive disease and provide a strong contextual framework for interpreting the clinical relevance of RDI-driven differences in response.

From a methodological perspective, these findings also carry implications for future trial design. Given the association between RDI and pCR demonstrated in this study, variability in chemotherapy delivery should be explicitly accounted for in neoadjuvant and perioperative trials in urothelial carcinoma. Failure to consider dose intensity may obscure true treatment effects, particularly in studies evaluating incremental benefits of novel agents added to chemotherapy backbones. Incorporation of RDI as a stratification factor or prespecified covariate could improve interpretability and reduce residual confounding related to heterogeneity in treatment exposure.

Clinically, these results highlight the importance of minimizing avoidable dose reductions and delays during neoadjuvant therapy. Renal dysfunction, performance status deterioration, treatment-related toxicity, and logistical factors frequently contribute to dose attenuation in routine practice.^33^ Optimized supportive care, proactive toxicity management, hydration strategies, and coordinated multidisciplinary oversight may therefore play a critical role in preserving dose intensity and maximizing the therapeutic potential of NAC.^34^ Given that pCR is achieved in only a minority of patients treated with standard chemotherapy, attention to treatment delivery may represent a clinically actionable aspect of perioperative management.^35^

This study has several strengths, including its large multicenter design, homogeneous chemotherapy exposure, and detailed reconstruction of cisplatin delivery using original administration records. The prespecified RDI threshold aligns with established dose-intensity literature, and the integration of clinically relevant covariates strengthens the robustness of the analyses. Nevertheless, limitations must be acknowledged. The retrospective design inherently introduces the possibility of residual confounding and selection bias. Patients maintaining higher RDI may have had more favorable baseline clinical characteristics, including better performance status, lower comorbidity burden, and improved treatment tolerance, which may not have been fully captured despite multivariable adjustment. Furthermore, clinician-driven treatment modifications and supportive care practices likely varied across participating centers. Detailed information regarding the specific causes of dose reductions or treatment delays, including prophylactic or therapeutic supportive care interventions such as G-CSF administration, was not uniformly available across centers. These factors may have influenced treatment delivery and the ability to maintain planned chemotherapy intensity. Accordingly, the observed associations should not be interpreted as evidence of causality.

Moreover, inclusion was restricted to patients who completed NAC and subsequently underwent radical cystectomy. Consequently, patients experiencing disease progression during NAC, significant clinical deterioration, or loss of surgical eligibility were not captured in the present analysis. This may have introduced selection bias toward a fitter population with more favorable disease biology, potentially limiting the generalizability of the findings and leading to overestimation of treatment efficacy. Patients discontinuing NAC before completion of at least three cycles were excluded; therefore, the impact of very early treatment interruption on pathological and survival outcomes could not be assessed.

Interestingly, both grade 1-2 and grade 3-4 neutropenia were more frequently observed among patients maintaining RDI ≥85%, potentially reflecting greater cumulative chemotherapy exposure among patients able to sustain planned treatment intensity. Similarly, low-grade nausea was more common in the RDI-high group, further supporting this interpretation. Conversely, severe renal and non-hematologic toxicities were not significantly different between groups. These findings further highlight the complex interplay between chemotherapy exposure, treatment tolerance, and clinician-driven dose modifications in retrospective analyses evaluating dose intensity.

Finally, follow-up duration remained relatively limited, with median OS and EFS not reached at the time of analysis. Consequently, survival findings should be considered exploratory and interpreted cautiously pending longer-term follow-up.

Despite these limitations, this study provides the first contemporary real-world evidence that preservation of cisplatin dose intensity during NAC is independently associated with improved pCR rate and associated with favorable survival outcomes in MIUC. In an era increasingly focused on therapeutic intensification through novel agents, these results underscore that optimization of chemotherapy delivery remains a fundamental and actionable component of high-quality neoadjuvant care.

## Conclusions

This study represents the first multicenter real-world analysis demonstrating that preservation of cisplatin RDI ≥ 85% during CG-NAC is independently associated with a higher probability of achieving pCR and may be associated with favorable survival outcomes in patients with MIUC. The persistence of this association after adjustment for established clinical and pathological prognostic factors suggests that preserved RDI may be associated with improved pathological response and favorable survival outcomes. These findings emphasize treatment delivery and management as a critical and potentially modifiable component of optimal perioperative management. Future prospective studies should evaluate whether strategies aimed at preserving chemotherapy intensity may improve pathological response and long-term outcomes.

## Supplementary Material

oyag256_Supplementary_Data

## Data Availability

The datasets generated during and/or analyzed during the current study are available from the corresponding author on reasonable request.
