# Supplementary material for: The impact of relative dose intensity on pathological complete response in neoadjuvant chemotherapy of muscle-invasive urothelial cancer: a multicenter retrospective study
Source: Oncologist. 2026 Jul 2;31(8):oyag256. doi: 10.1093/oncolo/oyag256 (PMC13395094; doi:10.1093/oncolo/oyag256)
Supplement: oyag256_Supplementary_Data [file oyag256_supplementary_data.docx]

Supplementary Materials.

The Impact of Relative Dose Intensity on Pathological Complete Response in Neoadjuvant Chemotherapy of Muscle-Invasive Urothelial Cancer: a Multicentre Retrospective Study

Giuseppe Neola^1,2#^, Fabiano Flauto^1#^, Carmine Caso^1^, Giuseppina Montuori^1^, Marco Maruzzo^3^, Eleonora Lai^3^, Giuseppe Luigi Banna^2,4^, Michele Maffezzoli^5^, Mimma Rizzo^6^, Francesco Massari^7^, Veronica Mollica^7^, Sarah Scagliarini^8^, Vincenza Conteduca^9^, Patrizia Giannatempo^10^, Alessandro Rametta^10^, Brigida Anna Maiorano^11^, Gaetano Facchini^12^, Edoardo Lenci^13^, Rosa Tambaro^14^, Francesco Grillone^15^, Davide Bosso^16^, Felice Crocetto^17^, Ciro Imbimbo^17^, Alberto Servetto^1^, Roberto Bianco^1^, Luigi Formisano^1^§.

1. Department of Clinical Medicine and Surgery, Federico II University, Naples, Italy.

2. Portsmouth Hospitals University NHS Trust, Portsmouth, United Kingdom.

3. Oncology Unit 3, Istituto Oncologico Veneto - IOV IRCCS, Padova, Italy.

4. Faculty of Science & Health, School of Pharmacy & Biomedical Sciences, University of Portsmouth, Portsmouth, UK.

5. Department of Medicine and Surgery, University Hospital of Parma - Medical Oncology Unit, University of Parma, Parma, Italy.

6. Medical Oncology Unit, Azienda Ospedaliera Universitaria Consorziale Policlinico di Bari, Bari, Italy.

7. Medical Oncology, IRCCS Azienda Ospedaliero-Universitaria di Bologna, Bologna, Italy.

8. UOC di Oncologia, Azienda Ospedaliera di Rilievo Nazionale Cardarelli di Napoli, Naples, Italy.

9. Unit of Medical Oncology and Biomolecular Therapy, Department of Medical and Surgical Sciences, University of Foggia, Policlinico Riuniti, Foggia, Italy.

10. Genitourinary Medical Oncology - Fondazione IRCCS Istituto Nazionale dei Tumori di Milano, Milan, Italy.

11. Department of Medical Oncology, IRCCS San Raffaele Hospital, Milan, Italy.

12. Oncology Unit, S. Maria Delle Grazie Hospital, Pozzuoli, Naples, Italy.

13. Medical Oncology Unit, Azienda Ospedaliera Ospedali Riuniti Marche Nord, Pesaro, Italy.

14. Uro-Gynecological Oncology, Istituto Nazionale Tumori - IRCCS - Fondazione G. Pascale, Naples Italy.

15. SOC Oncologia PO Pugliese-Ciaccio Azienda Ospedaliera Universitaria Renato Dulbecco, Catanzaro, Italy.

16. Medical Oncology Unit, Ospedale del Mare, 80147 Naples, Italy.

17. Department of Neurosciences, Reproductive Sciences and Odontostomatology, University of Naples Federico II, Naples, Italy.

#Co-first Authors.

§Corresponding Author.

| Adverse Event | RDI-High (N=219) | RDI-Low (N=111) | p-value |
| --- | --- | --- | --- |
| Asthenia | G1-G2: 87 (39.7%)  G3-G4: 10 (4.5%) | G1-G2: 35 (31.5%)  G3-G4: 4 (3.6%) | G1-G2: 0.150  G3-G4: 0.780 |
| Anemia | G1-G2: 115 (52.5%)  G3-G4: 18 (8.2%) | G1-G2: 64 (57.6%)  G3-G4: 13 (11.7%) | G1-G2: 0.414  G3-G4: 0.322 |
| Thrombocytopenia | G1-G2: 65 (29.6%)  G3-G4: 14 (6.3%) | G1-G2: 26 (23.4%)  G3-G4: 12 (10.8%) | G1-G2: 0.244  G3-G4: 0.194 |
| Neutropenia | G1-G2: 71 (32.4%)  G3-G4: 41 (18.7%) | G1-G2: 22 (19.8%)  G3-G4: 10 (9%) | G1-G2: 0.020  G3-G4: 0.024 |
| Thrombocytosis | G1-G2: 16 (7.3%)  G3-G4: 2 (0.9%) | G1-G2: 5 (4.5%)  G3-G4: 0 (0%) | G1-G2: 0.474  G3-G4: 0.552 |
| Hypertransaminasemia | G1-G2: 39 (17.8%)  G3-G4: 4 (1.8%) | G1-G2: 13 (5.9%)  G3-G4: 0 (0%) | G1-G2: 0.200  G3-G4: 0.302 |
| Hearing loss | G1-G2: 9 (4.1%)  G3-G4: 1 (0.4%) | G1-G2: 4 (3.6%)  G3-G4: 0 (0%) | G1-G2: 1.000  G3-G4: 1.000 |
| Renal Failure | G1-G2: 13 (5.9%)  G3-G4: 4 (1.8%) | G1-G2: 7 (6.3%)  G3-G4: 2 (1.8%) | G1-G2: 1.000  G3-G4: 1.000 |
| Vomiting | G1-G2: 27 (12.3%)  G3-G4: 2 (0.9%) | G1-G2: 9 (8.1%)  G3-G4: 0 (0%) | G1-G2: 0.269  G3-G4: 0.552 |
| Hand-Foot Syndrome | G1-G2: 5 (2.3%)  G3-G4: 0 (0%) | G1-G2: 1 (0.9%)  G3-G4: 0 (0%) | G1-G2: 0.668  G3-G4: NA |
| Thrombosis | G1-G2: 5 (2.3%)  G3-G4: 0 (0%) | G1-G2: 1 (0.9%)  G3-G4: 0 (0%) | G1-G2: 0.668    G3-G4: NA |
| Heart Failure | G1-G2: 2 (0.9%)  G3-G4: 0 (0%) | G1-G2: 0 (0%)  G3-G4: 0 (0%) | G1-G2: 0.552  G3-G4: NA |
| Nausea | G1-G2: 64 (29.2%)  G3-G4: 5 (2.3%) | G1-G2: 16 (14.4%)  G3-G4: 0 (0%) | G1-G2: 0.003  G3-G4: 0.172 |
| Mucositis | G1-G2: 34 (15.5%)  G3-G4: 4 (1.8%) | G1-G2: 16 (14.4%)  G3-G4: 1 (0.9%) | G1-G2: 0.872  G3-G4: 0.667 |
| Paresthesia | G1-G2: 37 (16.8%)  G3-G4: 5 (2.3%) | G1-G2: 12 (10.8%)  G3-G4: 1 (0.9%) | G1-G2: 0.189  G3-G4: 0.668 |

**Supplementary Table S1**. Treatment-related adverse events observed during neoadjuvant cisplatin-gemcitabine chemotherapy, stratified according to relative dose intensity (RDI ≥85% vs RDI <85%). Adverse events are reported according to CTCAE v5.0 and grouped as grade 1-2 and grade 3-4 toxicities. P-values were calculated using Fisher’s exact test comparing the frequency of grade 1-2 and grade 3-4 toxicities between RDI groups. P-values were not estimable when no events occurred in either group.


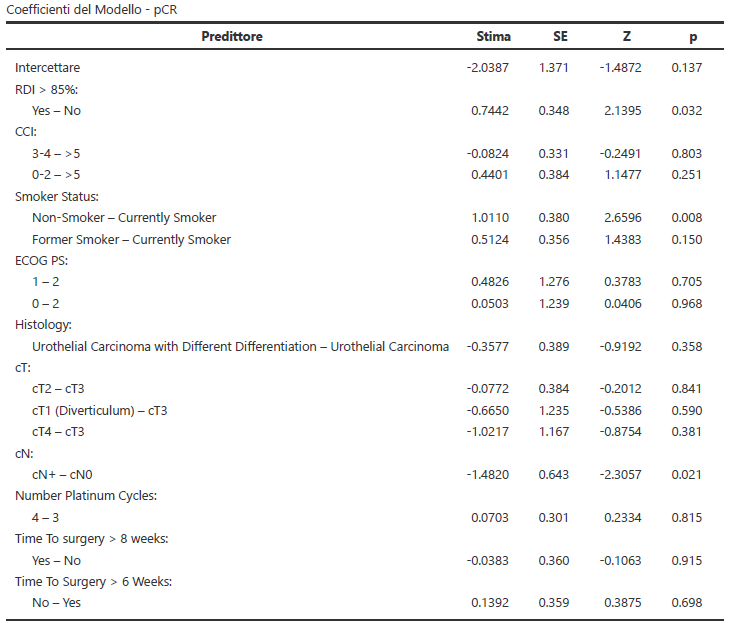


**Supplementary Table S2.** Multivariable logistic regression evaluating the association between RDI and pCR, adjusted for baseline clinical and pathological covariates.

**
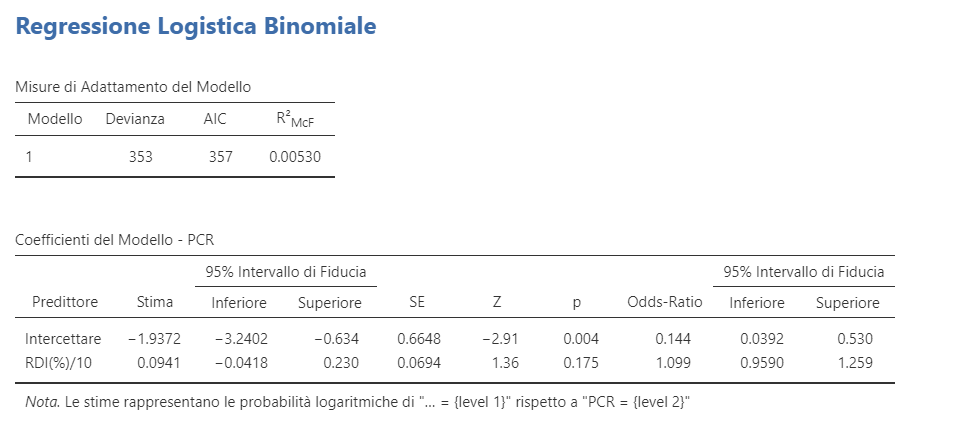
**

**Supplementary Table S3**. Exploratory sensitivity analysis evaluating the association between relative dose intensity (RDI) treated as a continuous variable and pathological complete response (pCR). Logistic regression analysis was performed using RDI expressed per 10% increase.

| Subgroup | RDI-High pCR | RDI-Low pCR | OR | 95%CI | p-value |
| --- | --- | --- | --- | --- | --- |
| ECOG 0 | 55/188 (29.3%) | 12/64 (18.8%) | 1.79 | 0.89-3.62 | 0.105 |
| ECOG 1-2 | 7/24 (29.2%) | 5/23 (21.7%) | 1.48 | 0.39-5.58 | 0.740 |
| CCI 0-2 | 13/41 (31.7%) | 9/30 (30.0%) | 1.08 | 0.39-3.01 | 1.000 |
| CCI ≥3 | 41/141 (29.1%) | 8/50 (16.0%) | 2.15 | 0.93-4.98 | 0.089 |

**Supplementary Table S4.** Sensitivity analyses evaluating the association between preservation of relative dose intensity (RDI ≥85%) and pathological complete response (pCR) across clinically relevant subgroups stratified by ECOG performance status and Charlson Comorbidity Index. Odds ratios (ORs), 95% confidence intervals (CIs), and p-values were calculated using Fisher’s exact test.

**
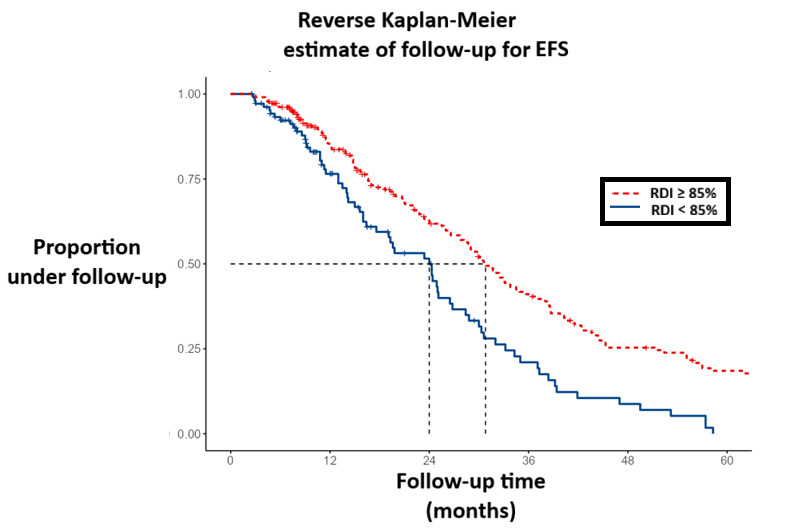
**

**Supplementary Figure S1.** Reverse Kaplan-Meier curves illustrating the distribution of follow-up time for the EFS cohort (N=320) analysis, stratified by RDI. The y-axis represents the proportion of patients remaining under follow-up over time. The horizontal dashed line indicates the 50% level, corresponding to the median follow-up, while vertical dashed lines mark the median follow-up time for each RDI group.

**
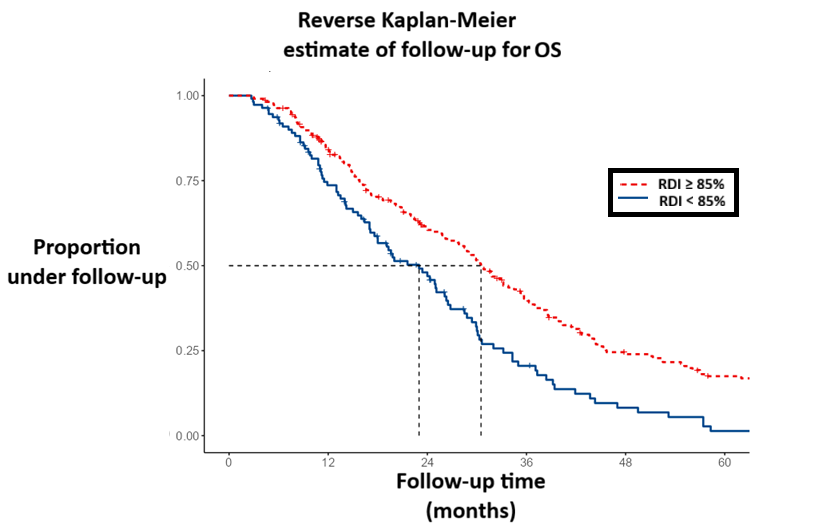
**

**Supplementary Figure S2.** Reverse Kaplan-Meier curves illustrating the distribution of follow-up time for the OS cohort (N=330) analysis, stratified by RDI. The y-axis represents the proportion of patients remaining under follow-up over time. The horizontal dashed line indicates the 50% level, corresponding to the median follow-up, while vertical dashed lines mark the median follow-up time for each RDI group.
